# The incompletely fulfilled promise of embryo transfer in cattle—why aren’t pregnancy rates greater and what can we do about it?

**DOI:** 10.1093/jas/skaa288

**Published:** 2020-11-03

**Authors:** Peter J Hansen

**Affiliations:** Department of Animal Sciences, D.H. Barron Reproductive and Perinatal Biology Research Program, and Genetics Institute, University of Florida, Gainesville, FL

**Keywords:** cattle, embryo transfer, fertility, in vitro fertilization

## Abstract

Typically, bovine embryos are transferred into recipient females about day 7 after estrus or anticipated ovulation, when the embryo has reached the blastocyst stage of development. All the biological and technical causes for failure of a female to produce a blastocyst 7 d after natural or artificial insemination (**AI**) are avoided when a blastocyst-stage embryo is transferred into the female. It is reasonable to expect, therefore, that pregnancy success would be higher for embryo transfer (**ET**) recipients than for inseminated females. This expectation is not usually met unless the recipient is exposed to heat stress or is classified as a repeat-breeder female. Rather, pregnancy success is generally similar for ET and AI. The implication is that either one or more of the technical aspects of ET have not yet been optimized or that underlying female fertility that causes an embryo to die before day 7 also causes it to die later in pregnancy. Improvements in pregnancy success after ET will depend upon making a better embryo, improving uterine receptivity, and forging new tools for production and transfer of embryos. Key to accelerating progress in improving pregnancy rates will be the identification of phenotypes or phenomes that allow the prediction of embryo competence for survival and maternal capacity to support embryonic development.

## Introduction—the Opportunity to Enhance Pregnancy Outcomes after Embryo Transfer

The ability to achieve pregnancy through the transfer of an embryo into the reproductive tract of a cyclic female, achieved for the first time in 1891 using the rabbit but not translated to routine practice in cattle until the 1970s (Betteridge, 2003), has been an extraordinary scientific success. Embryo transfer (**ET**) has not only helped illuminate fundamental processes of reproductive biology but has also become a tool for genetic selection of domestic animals, an essential adjunct for many biotechnologies, such as somatic cell nuclear cloning, gene editing, and the preservation and transport of germplasm, an established therapy for infertility in the human, and a method for propagation of endangered species.

ET is an effective means for facilitating births in humans because it bypasses roadblocks to successful pregnancy caused by errors in ovulation, oocyte development and maturation, fertilization, and early embryonic development. Circumvention of early pregnancy failure also occurs when ET is performed in cattle. Typically, bovine embryos are transferred into recipient females about day 7 after estrus or anticipated ovulation, when the embryo has reached the blastocyst stage of development. Occasionally, less-developed morulae are transferred when embryos are particularly valuable or there is a shortage of blastocysts. All the biological and technical causes for failure of a female to produce a blastocyst 7 d after natural or artificial insemination (**AI**) are avoided when a blastocyst-stage embryo is transferred into the female. It is reasonable to expect, therefore, that pregnancy success would be higher for ET recipients than for inseminated females.

As will be shown in this review, that expectation is not usually met, regardless of whether the transferred embryo was produced in vivo by superovulation or in vitro. The exception is for situations in which female fertility is very low because of heat stress or because the animal is persistently infertile (i.e., a repeat-breeder female). In the absence of these infertility factors, the percent of cows pregnant after embryo transfer (**P/ET**) is generally the same as the percent of cows pregnant after artificial insemination (**P/AI**) or only slightly higher. Thus, despite the avoidance of hazards to a successful pregnancy caused by errors in insemination procedures, fertilization failure, or embryonic death before the blastocyst stage, the bovine female is not much more likely to become pregnant than if she was bred by AI.

The conclusion is that either one or more of the techniques required for ET, including production, harvesting, or transfer of the embryo, have not yet been optimized or that underlying female fertility that causes an embryo to die before day 7 also causes it to die later in pregnancy. Thus, despite the many research advances that have made ET a procedure that can be performed routinely in many agricultural settings, the process has still not been perfected.

The purpose of this review is to compare the effectiveness of ET vs. AI for achieving sustainable pregnancies, discuss possible points in the ET process that could be suboptimal, and point out possible paths forward to increasing P/ET. When selecting papers to discuss, emphasis is placed on experiments in which 100 or more cows per treatment were enrolled. Papers based on smaller numbers of cows are also discussed when larger data sets are not available. The focus is on ET vs. AI because the literature does not include comparisons of ET with natural mating. The goal of writing the paper is to stimulate additional research to improve pregnancy outcomes after ET in cattle.

## Sources of Pregnancy Failure Avoided Using Embryo Transfer

Exclusive of technical errors, a cow inseminated at estrus can fail to become pregnant with a viable embryo at day 7 of development either because of anovulation, fertilization failure, or early embryonic death. Estimates of the incidence of anovulation at estrus in lactating dairy cows range from 3.4% to 6.7% (López-Gatius et al., 2005; Valenza et al., 2012; Burnett et al., 2018) except during heat stress when the incidence has been reported to increase to 12.4% (López-Gatius et al., 2005). Estimates of the incidence of ovulation failure in beef cattle were 2% for taurine heifers (Diskin and Sreenan, 1980) and 14% for both non-suckled cows and heifers that were largely Brahman crosses (Warnick and Hansen, 2010). Based on a compilation of research articles, Wiltbank et al. (2016) estimated that 18% of oocytes were not fertilized in lactating cows that were artificially inseminated under thermoneutral conditions. Using a similar approach, Santos et al. (2004) estimated the rate of fertilization failure of 22% for nonlactating dairy cows, 0% for dairy heifers, 25% for lactating beef cows, 1.4% for nonlactating beef cows, and 12% for beef heifers. Fertilization failure was estimated at 24% for beef cows and heifers by Warnick and Hansen (2010) and 5% to 11.5% for beef heifers (Diskin and Sreenan, 1980; Carter et al., 2008). The percent of zygotes that become nonviable before day 6 to 8 (based on the stage of development and morphological criteria) were estimated at 39% for lactating dairy cows under thermoneutral conditions (Wiltbank et al., 2016), 26% for nonlactating dairy cows, 28% for dairy heifers, 23% for lactating beef cows, and 19% for nonlactating beef cows, (Santos et al., 2004) and 0% to 13% for beef heifers (Diskin and Sreenan, 1980; Santos et al., 2004; Carter et al., 2008). 

**Figure 1.**
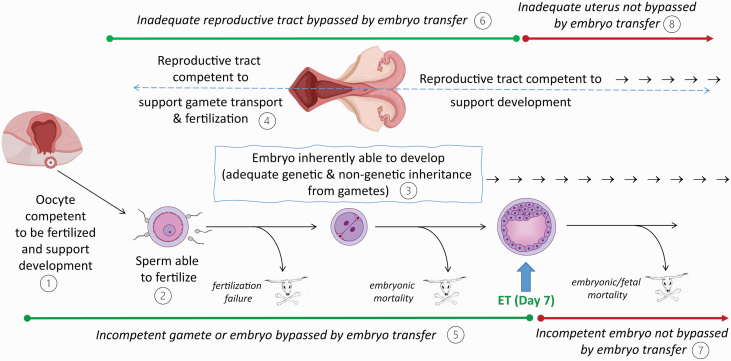
Requirements for a successful pregnancy prone to failure including those that can be alleviated by ET and those that cannot. Birth of a live, healthy calf requires ovulation of an oocyte capable of being fertilized and supporting the development of the resultant embryo (1), deposition of sperm in the reproductive tract capable of fertilizing the oocyte (2), formation of an embryo with the genetic and nongenetic inheritance from the oocyte and sperm that allow it to develop to term (3), and a reproductive tract competent to support gamete transport, fertilization, and development of the conceptus to term (4). For any given mating opportunity experienced by a female, one or more of these requirements can be lacking so that pregnancy is either not established or subsequently fails. Transfer of an embryo into the uterus at ~ day 7 of development can eliminate pregnancy failure caused by problems leading to fertilization failure or early embryonic mortality, including those caused by intrinsic defects in the gametes or embryo (5) as well as an inadequate reproductive tract (6). Transfer of an embryo does not prevent pregnancy losses caused by the inherent inability of the transferred embryo for development to term (7) or by the inability of the reproductive tract to support development after day 7 (8).

Except for ovulation failure, which will prevent pregnancy in all cases, biological causes for failure of a female to produce a blastocyst at 6 to 8 d after insemination are avoided when a blastocyst-stage embryo is transferred into the female. This point is illustrated in Figure 1, which shows the processes required for a successful pregnancy (i.e., one in which a healthy, neonatal animal is born) as well as potential causes of pregnancy failure that can be alleviated by ET. While not all oocytes used for the production of embryos for ET are capable of being fertilized and supporting the development of the resultant zygote to the blastocyst stage, the embryo selected for transfer is derived from an oocyte that has those properties. Similarly, while not every cow has a reproductive tract competent to support sperm survival, fertilization, and development of the early embryo, the embryo selected for transfer has been in an environment in which those processes were successful. Indeed, pregnancy rate at day 7 can be as high as 100% for ET recipients, provided a live embryo is successfully transferred into the uterus.

ET will not prevent all biological sources of pregnancy loss. Many blastocysts remain inherently incapable of developing to term, either because of chromosomal abnormalities, inheritance of genes that are detrimental to survival, or cytoplasmic inheritance from the oocyte being inadequate to support a sustained development. Through analysis of experiments in which cows either received two embryos or received an embryo and were inseminated, McMillan (1998) estimated that ~30% of embryos produced in vivo are inherently unable to develop to term. Furthermore, many cows that are recipients possess a reproductive tract that will not support development to term. McMillan (1998) estimated this percent as ~50% to 60%. Thus, even the most competent blastocyst transferred into a female could die if the maternal environment is inadequate.

There are also technical causes of failure to achieve pregnancy after insemination or ET. For AI, for example, females could be inaccurately diagnosed as in estrus, semen could be improperly stored, or the procedure for insemination could be practiced incorrectly. Technical errors also occur for ET such as improper handling of the embryo before transfer or deposition of the embryo in the wrong location in the reproductive tract. It is not clear whether technical problems are more prevalent for AI or ET although, under current commercial practice, ET practitioners often have more technical and academic education than most AI technicians.

## Comparisons of Pregnancy Success Following AI and ET

**Figure 2.**
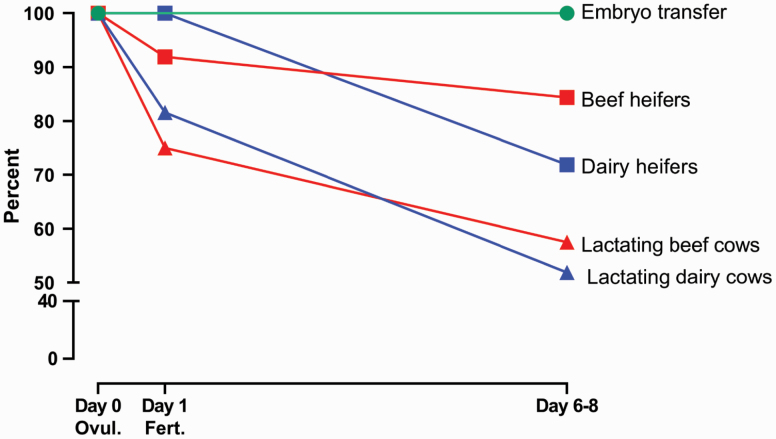
Effect of type of female and reproductive technique (ET using a fresh embryo vs. AI) on the percent of females eligible for pregnancy at ovulation (day 0) and percent pregnant at fertilization (day 1) and blastocyst formation (day 6 to 8). Data for beef heifers were calculated by summing results from two experiments (Carter et al., 2008; Diskin and Sreenan, 1980) and data for lactating dairy cows are from a compilation of studies by Wiltbank et al. (2016). Other sources of data were Sartori et al. (2002) for dairy heifers and Breuel et al. (1993) for lactating beef cows. Note that the estimate of 100% for pregnancy/ET assumes that only live embryos were transferred and that the embryo was correctly positioned during the transfer process. Values would be lower if some embryos transferred were dead or placed incorrectly.

Given the wastage in opportunities for a female to become pregnant, one would expect that pregnancy success in cattle would be greater following ET than following AI. The magnitude of the difference in pregnancy rate at day 6 to 8 of pregnancy between females receiving a fresh embryo via ET vs. for inseminated females is shown in Figure 2. Depending on the physiological status of the female, about 84% of inseminated beef heifers (Diskin and Sreenan, 1980; Carter et al., 2008) and 72% of dairy heifers (Sartori et al., 2002) would be pregnant at day 6 to 8 but only about 58% of lactating beef cows (Breuel et al., 1993) and 52% of lactating dairy cows (Wiltbank et al., 2016). These values compare to an expected P/ET of as high as 100% following transfer, provided a live embryo is successfully placed in the correct position in the uterus. Transfer of a dead embryo or errors in the transfer procedure would lower the expected pregnancy rate to less than 100%. In any case, it is to be anticipated that more ET recipients would be pregnant at later stages of gestation than for inseminated animals, particularly for lactating dairy cows. This is not usually the case, though.

One consideration must be made when comparing P/ET and P/AI. ET recipients only receive an embryo after the diagnosis of a corpus luteum (**CL**) by rectal palpation or ultrasonographic analysis of the reproductive tract. Thus, cows that do not ovulate do not receive an embryo and are removed from calculations of pregnancy success. For P/AI, however, pregnancy success is usually calculated based on all animals inseminated, even if they failed to ovulate. The resulting bias in pregnancy outcomes when comparing ET and AI should be adjusted for when assessing the two techniques.

The only comparisons of ET and AI in terms of pregnancy outcomes in the literature have involved lactating dairy cows. For some of these studies, heat stress was present for all or part of the observational period and, as will be discussed below, this phenomenon complicates comparisons of the two techniques. There are, however, three studies in the literature where the effectiveness of ET for achieving pregnancy could be compared to AI in the absence of extensive heat stress. In each of these experiments, there was little difference in pregnancy outcomes between AI and ET after adjusting data for ovulation failure (Table 1).

**Table 1.** Comparison of pregnancy outcomes to ET and AI (after adjustment for ovulation failure) for experiments with lactating Holsteins in which extensive heat stress was not present^1^

Method	Day of pregnancy diagnosis	Number of cows	Percent pregnant	Notes	Reference
TAI	25 to 32	238	31.1	Wisconsin	Sartori et al. (2006)
TET, in vivo, 21.5% fresh, and 78.5% frozen		250	34.4		
AI		5,405	35.7	Brazil: cool months of June, July, & August	Rodrigues et al. (2007a)
ET, in vivo, fresh, and frozen		1210^2^	33.5		
TAI	Day 30, based on pregnancy- associated glycoproteins detection	1767	47.7	California: November to May	Oliveira et al. (2019a)
TET, in vivo fresh		329	55.3		
TET, in vivo frozen		322	47.3		
TET, in vitro fresh		456	43.0		

^1^Data were adjusted to so that cows assigned to the ET group that did not have a CL were considered nonpregnant (Sartori et al., 2006; Oliveira et al., 2019a) or, for Rodrigues et al. (2007a), by multiplying pregnancy rate in the ET group by 0.80 [assuming 20% of cows did not have a CL and would not have been pregnant if an ET was actually performed; estimate of rate of CL detection based on P.S. Baruselli (personal communication)].

^2^The number of cows pregnant was 507. If one assumes 20% of cows were excluded because of no CL, the number of animals enrolled would be 1,512.

**Figure 3.**
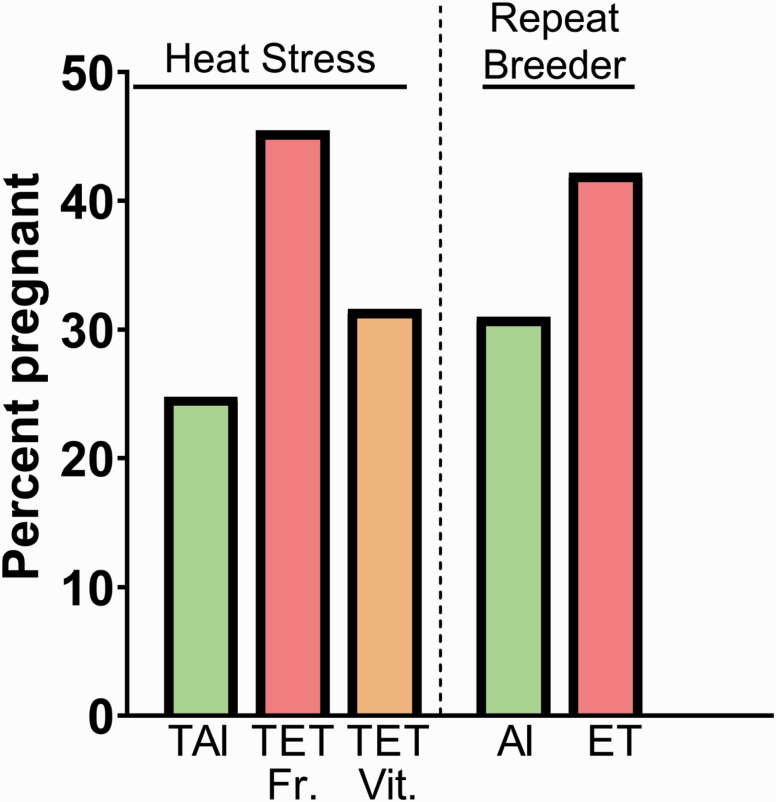
Two cases where ET increases pregnancy rate as compared with AI—during heat stress and in repeat-breeder cows. The data for the heat stress experiment are from Stewart et al. (2011) and represent P/AI or P/ET at day 40 after estrus for lactating dairy cows in the summer in Texas that were successfully synchronized using an ovulation synchronization protocol (TAI or TET). Both AI and ET involved the use of X-sorted semen. The total number of cows was 485. Embryos were produced in vitro and either transferred fresh (Fr.) or vitrified (Vit.). The data for the study with repeat-breeder cows are from Rodrigues et al. (2007b) and represent data for cows that were either AI at estrus or received an embryo produced by superovulation 7 d after estrus. The data shown are AI or ET results for the months of June to August (*n* = 1,518 AI and *n* = 967 ET) for cows that had been inseminated unsuccessfully at least three times previously. The original pregnancy rate for the AI group (24.8%) has been adjusted for this paper by multiplying by 0.8 to account for the approximately 20% of AI cows without a CL.

There are two situations in which ET does consistently improve fertility as compared with AI. The first is during periods of heat stress. The benefits of ET for improving fertility in heat-stress cows have been reviewed extensively (Hansen, 2019; Baruselli et al., 2020). Most of the infertility caused by heat stress is because of damage to the oocyte and early embryo. By the time an embryo is transferred, at the blastocyst stage, the embryo has acquired resistance to maternal thermal stress. As a result, there can be a large improvement in pregnancy rate using ET in summer (see Figure 3 for an example), and there is little or no seasonal variation in P/ET even in hot climates (Hansen, 2019; Baruselli et al., 2020).

The other situation in which ET can improve pregnancy outcomes is for the repeat-breeder cow. These cows are usually defined as those that have been inseminated three or more times without the establishment of pregnancy. Several retrospective and prospective studies have shown that pregnancy success after ET in repeat-breeder cows can be greater than for AI (Tanabe et al., 1985; Son et al., 2007; Block et al., 2010). Selected results from the largest study, in which results from 5,693 AI and 3,858 ET were analyzed (Rodrigues et al., 2007b), are illustrated in Figure 3. The reason why ET improves fertility is unclear and causes of the repeat-breeder syndrome are likely to differ between individual cows and between herds of cows. Some causes of the repeat breeder syndrome, such as reduced oocyte quality (Kurykin et al., 2011; Sood et al., 2017), would be expected to be alleviated by ET, whereas other causes, such as alterations in endometrial function (Katagiri and Takahashi, 2004), would be reversible by ET only if the disorder was resolved after the day of transfer.

There are several reports from Brazil using data collected throughout the year where P/ET was higher than P/AI (Demetrio et al., 2007; Vasconcelos et al., 2011a; Pereira et al., 2016). Most recently, for example, Pereira et al. (2016) performed a retrospective analysis to compare timed AI (**TAI**) and timed ET (**TET**) for 13 dairies. Embryos were produced in vitro and transferred fresh. Only cows that ovulated to the ovulation synchronization protocol were included. Pregnancy rate at day 60 was lower (*P* < 0.01) for TAI (31%; 1,709/5,430) than for TET (34%; 685/2,003). While statistically significant, the magnitude of the difference in pregnancy success between TAI and TET was much less than one would expect based on the expected differences in embryonic survival before day 7.

## Causes of Pregnancy Loss Associated with ET

The successful use of ET to improve fertility during heat stress and for repeat-breeder cows points out the promise of this procedure as an assisted reproduction technology for improving fertility. That the benefits of ET for increasing pregnancies are not more widespread is an indication that either the technology for ET is not yet perfected or that there are causes of infertility that cannot be corrected by bypassing pregnancy losses through the first 7 d of gestation. Given that pregnancy rate at day 7 can be as high as 100% for ET recipients and is only about 50% to 60% for lactating cows (Figure 2), the day 7 embryo transferred into a recipient female is at a much higher risk for death before first pregnancy diagnosis at ~ day 28 of gestation than is the day 7 embryo produced by AI. There are also indications that pregnancy loss after initial pregnancy diagnosis is greater for ET than AI, for embryos produced both in vitro (Stewart et al., 2011) and in vivo (Demetrio et al., 2007). In other cases, however, there was no difference between pregnancy loss for AI vs. transfer of an embryo produced in vitro (Pereira et al., 2016) or in vivo (Sartori et al., 2006).

In reliability engineering, a key aspect of increasing reliability is to identify and correct the causes of failures (O’Connor, 2002). Achieving the goal of increasing the reliability of ET for pregnancy success will depend on the identification of causes of failure of an embryo transferred into a recipient to develop to term. This section of the review will focus on known and possible causes of embryo loss between the day of transfer and first pregnancy diagnosis due to the embryo, the recipient, and technical issues regarding ET.

### Pregnancy losses due to the embryo

There are many permutations in the practice of ET. Broadly speaking, however, embryos for transfer are either produced in vivo (multiple ovulation embryo transfer [**MOET**]) or in vitro (in vitro produced [**IVP**]) and either transferred fresh or after cryopreservation. Embryos are produced in vivo by treating cows with follicle-stimulating hormone (**FSH**) to induce multiple ovulations followed by insemination at estrus or the anticipated time of ovulation, and recovery of embryos by flushing the uterus at day 7 after estrus (Mikkola et al., 2019). Embryos are produced in vitro by aspiration of oocytes from follicles (usually by ultrasound-guided transvaginal aspiration but sometimes by harvesting oocytes from ovaries ex situ), inducing nuclear and cytoplasmic maturation of the oocytes in culture, incubating the matured oocytes with capacitated sperm to achieve fertilization, and then culturing the resultant zygotes to the morula or blastocyst stage for transfer (Tríbulo et al., 2019).

#### The embryo produced in vitro

On average, the IVP blastocyst has many characteristics that differ from that of a blastocyst produced in vivo by superovulation, including oxygen consumption, accumulation of intracellular lipid, other ultrastructural features, gene expression, and DNA methylation (reviewed by Hansen, 2020). The incidence of abnormal chromosomes is also higher than for MOET embryos (Viuff et al., 1999; Dieleman et al., 2002; Tšuiko et al., 2017). As many as 72% of IVP blastocysts were reported to exhibit mixoploidy (Viuff et al., 1999). Following transfer, there are reports of compromised trophoblast elongation, loss of the embryonic disk, changes in placental function, and dysregulation of fetal development (reviewed by Ealy et al., 2019). Most important for the current discussion, a smaller percent of transferred IVP embryos result in pregnancies than when MOET embryos are transferred (Ealy et al., 2019). Clearly, technical procedures to produce a transferrable embryo in vitro have not yet been optimized. Given the increasing use of IVP embryos in cattle production as compared with MOET embryos (Hansen, 2020), this is a missed opportunity.

#### Abnormal development of the embryo produced by superovulation

Most experiments performed to define abnormalities of the IVP embryo involve comparisons with the embryo produced in vivo using superovulation. Yet, there is reason to suppose that the MOET embryo is also abnormal when compared with an embryo recovered from a non-stimulated female. Superovulation can cause changes in the follicle and follicular fluid (Dias et al., 2013a; Santos et al., 2018; Fontes et al., 2019), oocyte (Chu et al., 2012), oviduct (Fontes et al., 2019), and endometrium (Forde et al., 2012). The effects of superovulation on the reproductive tract could reflect the elevation in circulating concentrations of progesterone associated with the procedure (Forde et al., 2012; O’Hara et al., 2012). Elevated concentrations of estradiol in the oviduct (Fontes et al., 2019) and blood plasma (Santos et al., 2018) can also occur for cows superovulated with combined treatment with FSH and equine chorionic gonadotropin. Possibly, there are direct effects of FSH on the reproductive tract because the oviduct and uterus express gonadotropin receptors and can be modified by luteinizing hormone and human chorionic gonadotropin (Ziecik et al., 2005). Forde et al. (2012) found that the number of genes whose expression in the endometrium at day 7 of pregnancy was altered by superovulation was much higher than the number found to be altered in expression by supplemental progesterone treatment, either because progesterone concentrations are so much higher after superovulation or because of other physiological changes induced by the superovulation regimen.

Not surprisingly, given the difficulty in collecting embryos produced from non-stimulated females, there has been no direct comparison of the competence to establish pregnancy for blastocysts produced by superovulation vs. from a non-stimulated female. Studies of gene expression differences suggest that the two types of embryos may not be equivalent, however. Analysis of a limited set of expression of eight genes indicated that transcript abundance for *GRB10*, an adapter protein for insulin and insulin-like growth factor receptors, was highest in IVP blastocysts, intermediate in MOET blastocysts, and lowest in blastocysts produced by natural mating after spontaneous estrus (Mundim et al., 2009). Using microarray analysis, Gad et al. (2011) identified 454 genes that were differentially expressed between MOET embryos that developed in the uterus until day 7 vs. MOET embryos that were transferred to the oviduct of a non-stimulated heifer at day 2 of development. Of the differentially expressed genes, 429 were more abundant in blastocysts that developed in superovulated females. These results, in which all embryos were produced by superovulation but where development took place in the uterus of a stimulated or non-stimulated donor, point to the importance of alterations in endometrial function caused by superovulation as a cause for altered characteristics of the blastocyst.

Though less frequent than for IVP embryos (Viuff et al., 1999; Tšuiko et al., 2017), there is a high incidence of chromosomal abnormalities in MOET embryos. A total of 25% of MOET blastocysts exhibited mixoploidy (Viuff et al.,1999), and 41.5% of MOET embryos collected at day 12 to 18 of pregnancy were mixoploid (Hare et al., 1980). The significance of mixoploidy for embryo survival is unknown. Pregnancies were achieved when mixoploid embryos collected from days 12 to 15 of pregnancy were transferred to recipients (Hare et al., 1980). It will be important to compare whether the incidence of chromosomal abnormalities in embryos produced by superovulation is any different than for embryos from non-stimulated females.

#### Cryopreservation

There are two general strategies for cryopreservation of bovine embryos. In the first, often called slow freezing or controlled-rate freezing, embryos are gradually mixed with a cryoprotectant mixture, loaded in a straw, seeded for ice crystal formation, gradually cooled at a slow (0.3 to 0.5 °C) programmed rate, and, when the temperature reaches between −30 and −65 °C, plunged into liquid nitrogen (Saragusty and Arav, 2011). For vitrification, the goal is to achieve cryopreservation of the embryo in a glass-like state to avoid the ice crystal damage associated with slow freezing. Vitrification is achieved by very rapid cooling in a small volume and with high concentrations of cryoprotectants (Saragusty and Arav, 2011).

There have been few experiments with large numbers of animals to compare the two approaches to cryopreservation. Pregnancy rates were similar for slow freezing and vitrification for MOET embryos (van Wagtendonk-de Leeuw et al., 1997) and for IVP embryos (Sanches et al., 2016). Slow freezing remains popular because embryos can be transferred directly, whereas the high concentrations of toxic cryoprotectants used for vitrification make washing of the embryo after thawing a necessity.

**Figure 4.**
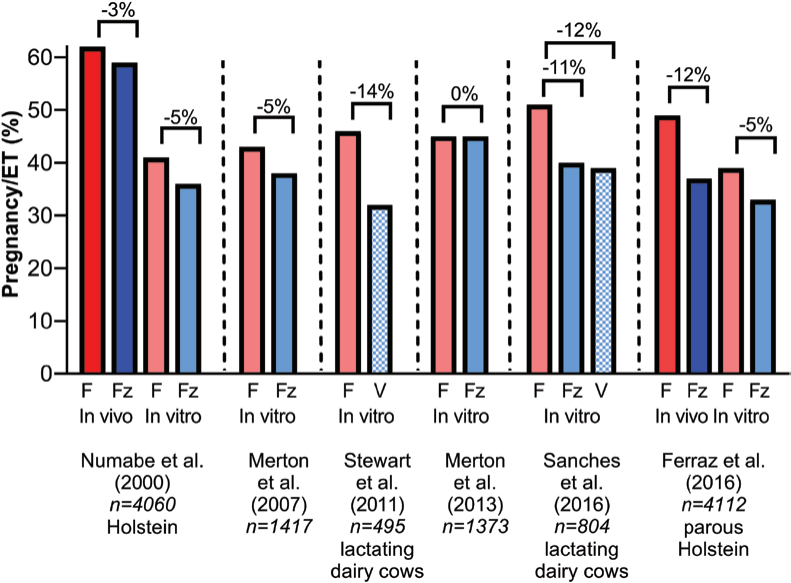
Comparison of pregnancy rates per ET for embryos produced in vivo (red and blue) and in vitro (light red and light blue) and that were transferred fresh (F; red or light red solid bars), frozen (Fz; blue or light blue solid bars), or vitrified (V; light blue hatched bars). The difference in pregnancy/ET between fresh and cryopreserved embryos is shown above each pair of bars. Data are from Numabe et al. (2000), Merton et al. (2007, 2013), Stewart et al. (2011), Sanches et al. (2016) and Ferraz et al. (2016).

Cryopreservation is associated with a reduction in P/ET. A summary of results from six large-scale studies comparing pregnancy results for recipients receiving fresh embryos vs. those that were either frozen or vitrified is presented in Figure 4. Across all studies, P/ET for cows receiving cryopreserved embryos was 7.4% points lower than for cows receiving fresh embryos. It is often stated that the IVP embryo is more sensitive to damage caused by cryopreservation than the MOET embryo. This view is not consistent with results from the two large-scale studies in which both types of embryos were evaluated (Figure 4). While overall pregnancy rates were higher when a MOET embryo was transferred, the magnitude of the reduction in P/ET caused by cryopreservation was either similar for embryos of both types (Numabe et al., 2000) or higher for MOET embryos than IVP embryos (Ferraz et al., 2016). The particular culture conditions used to produce embryos in vitro can affect the ability of IVP embryos to survive cryopreservation (Block et al., 2009; Paschoal et al., 2014), and some systems used for the production of IVP embryos do result in blastocysts with poor survivability to cryopreservation (Vieira et al., 2007; Estrada-Cortés et al., 2019).

There are also concerns in the industry that embryos of *Bos indicus* cattle are more sensitive to cryopreservation than *Bos taurus* embryos but the one large-scale study investigating this issue failed to detect a difference in P/ET between genetic types (Marinho et al., 2015). 

### Pregnancy losses due to the recipient

A poor uterine environment could not only lead to embryonic death before day 7 in an inseminated cow but, if the uterine environment continues to remain inadequate, could also cause the death of a healthy embryo transferred into the uterus at day 7. McMillan (1998) estimated that only about 40% to 50% of recipient females are capable of supporting the establishment of pregnancy. Other experiments in which embryos were repeatedly transferred to cows have revealed that there are subpopulations of highly fertile females that become pregnant after all, or nearly all, ET and other subpopulations that rarely or never become pregnant (McMillan and Donnison, 1999; Geary et al., 2016; Moraes et al., 2018). Further evidence for poor uterine receptivity as a cause of infertility was the finding that 14 of 37 (32.4%) recipients receiving five embryos failed to become pregnant to any embryo (Martins et al., 2018a). Examples of cow factors associated with poor P/ET are low body condition score (Mapletoft et al., 1986; Wallace et al. 2011), poor weight gain (Fernandes et al., 2016), lactation or parity in dairy cattle (Dochi et al., 1998; Hasler, 2001; Ferraz et al., 2016), temperament (Kasimanickam et al., 2018, 2019), and periparturient or postpartum disease (Ferraz et al., 2016; Ribeiro et al., 2016; Barbosa et al., 2018; Estrada-Cortés et al., 2019).

#### Does the embryo prepare the uterus to support its development?

**Figure 5.**
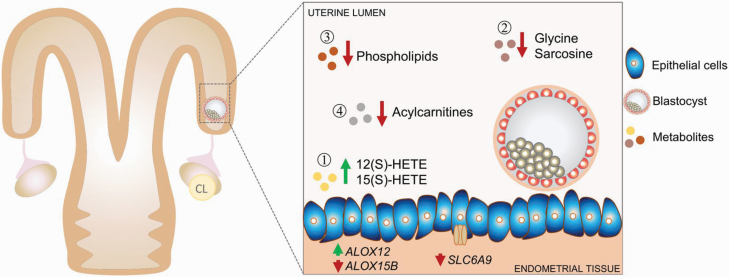
Representation of local effects of the bovine blastocyst on the endometrium at day 7 of pregnancy. Of 205 metabolites examined in uterine flushings from the cranial portion of the uterus ipsilateral to the side of ovulation, 22 differed between pregnant and nonpregnant cows. The lipoxygenase products 12(S) and 15(S)-hydroxyeicosatetraenoic acid (HETE) were higher in pregnant cows and the other 20, including specific phospholipids, acylcarnitines, glycine, and sarcosine, were lower. Expression of one lipoxygenase gene, *ALOX12*, was increased i n the uterotubal junction, whereas another, *ALOX15B,* was decreased. The decrease in glycine content of the uterine lumen was consistent with decreased expression of the glycine transporter *SLC6A9*. Consequences of local actions of the blastocyst on the endometrium for long-term function of the blastocyst remain to be determined. The figure is reproduced from Sponchiado et al. (2019) and is reproduced from *Scientific Reports* through a Creative Commons Attribution 4.0 International License.

ET would pose a specific problem to the establishment of a receptive uterine environment if embryo-induced changes in endometrial function occurring before day 7 of pregnancy are important for embryo survival after day 7. The embryo has a very observable effect on maternal physiology between day 15 and 17 of pregnancy when trophoblast-derived interferon-τ blocks luteolysis (Northey and French, 1980). Pregnancy can result when an embryo is transferred into the uterus as late as day 16 after estrus but not when the transfer is on day 17 (Betteridge et al., 1980). The release of interferon-τ is not, however, the first regulatory signal the embryo sends to the mother. Experiments in the sheep by Ashworth and Bazer (1989) showed that the day 6 embryo placed in the day 4 uterus can alter secretory protein synthesis by the endometrium in a manner that advances the uterine environment. The bovine embryo, too, can signal the mother and as early as day 7 of gestation. The local presence of the embryo causes changes in abundance of specific endometrial transcripts (Sponchiado et al., 2017) and in the metabolome of the uterine lumen (Sponchiado et al., 2019). An example of embryo-induced changes in the uterine fluid metabolome and associated changes in uterotubal gene expression is illustrated in Figure 5.

### Pregnancy losses due to the technique

Initially, ET was performed surgically. Nonsurgical transfer, involving transcervical placement of the embryo in the uterine horn ipsilateral to the CL (Boland et al., 1976), was a major advance because ET could be performed by a larger variety of personnel in a wider range of settings with less risk to the recipient. In some programs, pregnancy rates were similar for nonsurgical and surgical transfer (Mapletoft et al., 1986; Hasler, 2006). In others, however, higher pregnancy rates were achieved with surgical transfer (Hasler, 2006). It is difficult to make conclusions regarding the relative efficacy of the two approaches because of differences in technical proficiency amongst individuals and groups. In any case, it is worth considering whether the insults applied to the reproductive tract during nonsurgical transfer can compromise the recipient or embryo.

During the process of nonsurgical transfer, the reproductive tract is manipulated to guide the transfer instrument past the cervix and into the uterine horn. This process exposes the endocervix and endometrium to possible irritation or trauma caused by the transfer instrument. In addition, a small volume of a foreign fluid is introduced into the uterine lumen coincident with expulsion of the embryo. Another possible consequence of nonsurgical transfer is the uterine release of prostaglandin F_2α_ caused by the manipulation of the uterus *per rectum* (Wann and Randel, 1990).

That one or more of insults to the reproductive tract are potentially deleterious to pregnancy establishment is indicated by observations that the difficulty in passing the cervix and depositing the embryo in the uterus is negatively associated with P/ET (Jaśkowski et al., 2010; Kasimanickam et al., 2018, 2019; Alkan et al., 2020). Moreover, the physical characteristics of the transfer instrument have been reported to affect pregnancy success. The P/ET was higher when embryos were transferred using a steel instrument with a gold-plated tip and no plastic sheath than when using a instrument covered with plastic sheath (Jaśkowski et al., 2010). Treatment of recipients with flunixin meglumine, an inhibitor of prostaglandin F_2α_ synthesis, improved P/ET in several experiments (Purcell et al., 2005; Scenna et al., 2005; Kasimanickam et al., 2018, 2019).

Placement of a large volume of fluid (30 mL) can increase the accumulation of proteins in the uterine lumen and compromise embryo survival (Martins et al., 2018b). Whether a smaller volume of fluid such as that in a 0.25-mL ET straw also causes deleterious changes in the uterine fluid composition is unknown. Some media also contain antioxidants, buffers, allogeneic serum, and antibiotics that could possibly cause local changes in endometrial function. It is also not known whether embryonic survival is compromised by cotransfer of infectious agents. Viral transmission to recipient females via ET has been demonstrated recently (Haegeman et al., 2019), and it can be difficult to remove *Coxiella burnetti* (Alsaleh et al., 2014) and *Chlamydia abortus* (Pellerin et al., 2019) from IVP embryos. Signatures of bacterial contamination of media used for embryo production have been identified using mass spectrometry (Zampieri et al., 2013).

Fresh embryos may spend several hours in an ET straw until transferred into a recipient. Nothing is known about whether the embryo experiences changes in its physiology while in the straw that affect competence to establish pregnancy. Such a phenomenon must be considered; metabolism of mouse embryos changes within 3 h after removal from the reproductive tract and placement in culture (Lane and Gardner, 1998). Currently, we do not know the medium composition or the storage temperature that is optimal to maintain embryo competence for pregnancy, while it is held in a straw.

## Some Ways Forward

At the beginning of this review, the question was posed as to why pregnancy success after ET was not a great deal higher than what was achieved following AI, given the fact that ET bypasses pregnancy failures due to anovulation, fertilization failure, and early death of the embryo. It is evident from the literature that lower than expected P/ET can be ascribed to three general causes. First, techniques to produce an embryo for transfer often result in an embryo that has molecular or cellular characteristics that reduce competence for establishment of pregnancy after transfer. This is clearly the case for the IVP embryo and it may also be true to a lesser extent for an embryo produced by superovulation. Second, while it is true that ET can bypass early embryonic death (i.e., before day 7) caused by an inadequate reproductive tract environment, embryo death could occur after day 7 if the reproductive tract remains aberrant after transfer. A third cause of suboptimal outcomes of ET would be that the techniques utilized for the transfer process may not yet be optimized. Technical inefficiencies can lead to embryonic death.

Given these three general causes of failure of ET, there are many avenues of research that could lead to the development of practices and procedures that increase P/ET. A non-exhaustive description of some of these is discussed in this section.

### Making a better embryo

Much of the research on improvements in MOET and IVP is focused on increasing the yield of transferrable embryos per donor. Greater attention to the effect of embryo production protocols on the competence of the resultant embryos to establish pregnancy could lead to higher P/ET than is the case currently. Unfortunately, most academic laboratories do not have access to recipients in suitable numbers to evaluate embryo competence for establishing pregnancy. For this reason, partnerships between academia and commercial and government organizations involved in the transfer of embryos in the field are essential to advance our ability to improve embryo competence for pregnancy.

There are two pathways for making a better embryo—that for the MOET embryo and that for the IVP embryo. Focus on the IVP embryo may be more fruitful because embryo competence for pregnancy establishment is lower (Ealy et al., 2019), and there are probably more opportunities to manipulate the production system than for MOET. Moreover, transfer of IVP embryos is growing worldwide while that of MOET embryos is relatively stable (Hansen, 2020). If procedures to generate gametes from embryonic stem cells become a reality (Goszczynski et al., 2019), the use of IVP embryos will accelerate.

#### The IVP embryo

It is a reasonable expectation that the most competent embryos for pregnancy establishment will be derived from culture systems that either closely mimic conditions in vivo for oocyte maturation, fertilization, and embryonic development or utilize alternative solutions to place embryos in the same physiological state as would occur in vivo. There have been important changes in culture systems since the first calf from an IVP embryo was produced in 1981 (Brackett et al., 1982), including modifications of procedures for oocyte maturation, fertilization, and embryo culture (see Parrish, 2014; Sirard, 2018; and Ferré et al., 2020 for some historical perspective). Even today, however, culture systems differ in many ways from the situation in vivo, including the substratum embryos reside on (plastic instead of epithelium), availability of energy substrates, amino acids, micronutrients and cell-signaling molecules, and fluid dynamics around the embryo.

One indicator that culture systems are suboptimal is the fact that the yield of blastocysts is greater when embryos are cultured either in groups or in restricted spaces with small volumes (O’Doherty et al., 1997; Vajta et al., 2000; Oliveira et al., 2019b). Requirement for a high density of embryos is an indicator that the embryos add important nutrients or cell-signaling molecules to the culture medium or that embryos remove molecules that are toxic to the embryo. Another indicator of inadequacy of culture systems is the observations that IVP embryos differ metabolically from embryos developing in vivo, including increased oxygen consumption (Lopes et al., 2007) and amino acid turnover (Sturmey et al., 2010).

Accurate determination of the metabolome of the oviduct and uterus and of the nutritional requirements of the oocyte and embryo might lead to the development of more appropriate culture media for IVP. Culture of embryos in a medium in which the amino acid concentrations reflected those in oviduct fluid increased blastocyst yield and freezability as compared with a standard amino acid mix (Li et al., 2006). In the same paper, one of the two media based on amino acid concentrations in the uterine fluid was effective in supporting blastocyst development; the other medium was inhibitory to development. The addition of the methyl donor choline to cultured embryos also had a slight positive effect on blastocyst yield when added at a concentration of 4 μM (Estrada-Cortés et al., 2020). The [Mg^2+^] in embryo culture medium has likewise been reported to affect the percent of oocytes becoming blastocysts (An et al., 2019).

The bovine embryo can take up proteins in the culture medium and use them as an amino acid source (Thompson et al., 1998). Most culture media used for the production of bovine embryos in vitro add either serum or bovine serum albumin (**BSA**). There is evidence in the sheep that serum can alter the developmental program of the embryo to increase the incidence of large offspring syndrome (Rooke et al., 2007). BSA derived from biological sources is not a purified substance and even contains growth factors that can alter embryonic function (Gómez et al., 2017). Presence of BSA in the medium can also reduce cryopreservation (Block et al., 2009; Murillo-Ríos et al., 2017). One alternative that has not been explored in cattle is the use of a recombinant protein source. In the human, recombinant human serum albumin is an effective substitute for albumin purified from blood (Bungum et al., 2002) and is less likely to introduce non-defined molecules into the culture medium. It should also be recognized that BSA is a biologically active molecule that can bind a host of small molecules, including long-chain fatty acids, bilirubin, tryptophan, thyroxine, hemins, heavy metals, and vitamins (Kragh-Hansen et al., 2002; Bteich, 2019). Other proteins may provide a better environment for the embryo than BSA.

In vivo, the embryo is regulated by cell signaling molecules in oviductal and uterine fluid called embryokines. Many cell-signaling molecules have been identified that can regulate embryonic development in vitro (Hansen and Tríbulo, 2019). Some of these, including the cytokine colony-stimulating factor 2 (Loureiro et al., 2009; Denicol et al., 2014), the WNT signaling inhibitor dickkopf-1 (Denicol et al., 2014), and, during heat stress, the growth factor insulin-like growth factor 1 (Block and Hansen, 2007), can act on the IVP embryo to increase competence to establish pregnancy after transfer into females. More work to identify the array of embryokines produced by the oviduct and embryo and to understand how they act in combination to regulate characteristics of the blastocyst could lead to higher P/ET.

It is well established that the period of oocyte growth and maturation is important for the capacity of the resultant embryos to develop to the blastocyst stage (Rizos et al., 2002; Dias et al., 2013b; Gilchrist et al., 2016). Little has been done experimentally to determine whether modifying conditions of oocyte maturation can alter the competence of the transferrable embryo for establishing pregnancy. Addressing this question could also prove fruitful for increasing P/ET.

**Figure 6.**
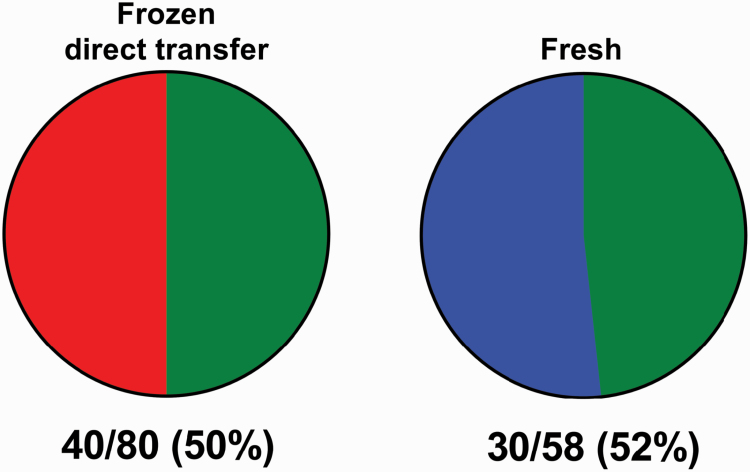
Results from a report of a new system for direct transfer of frozen embryos without loss in pregnancy success as compared with fresh embryos. Shown are pregnancy data in which heifers received either a fresh or frozen embryo. Numbers at the bottom represent the fraction and percent of cows pregnant after transfer. Embryos were produced in vitro and were cultured with BSA until day 6 and then without protein to day 7. Blastocysts at day 7 were transferred to heifers either fresh or after freezing in a medium using a synthetic protein substitute called CRYO3. Frozen embryos were thawed and then transferred directly without removing cryoprotectants. Results are from Gómez et al. (2020).

There are also good prospects that modifications of media for IVP can enhance cryosurvival of embryos. Recently, Gómez et al. (2020) reported that IVP embryos cultured with BSA to day 6 and then without protein to day 7 achieved a pregnancy rate following freezing and direct transfer that was similar to that following transfer of fresh embryos (Figure 6). Freezing was performed in ethylene glycol with a synthetic protein substitute called CRYO3.

#### The MOET embryo

MOET will continue to be an important method for producing embryos for transfer because few laboratory resources are required and P/ET is superior to that achieved with IVP embryos (Ealy et al., 2019). Research continues to improve superovulatory protocols with the goal of increasing blastocyst yield (Bó and Mapletoft, 2020), and some of these methods may also affect embryo competence for pregnancy. In addition, new preparations of gonadotropins, including a long-acting FSH (Sanderson and Martinez, 2020), could result in novel protocols that change the type of follicle recruited or limit disruption in reproductive tract function.

Regulation of the reproductive tract environment of embryo donors may also result in embryos with superior ability to establish pregnancy. One approach might be nutraceutical. Pregnancy rates were higher when MOET embryos were produced from cows fed a supplement rich in linoleic acid and containing β-carotene than when embryos were from control cows (Takahashi et al., 2013). Another approach might be hormonal. The P/ET was greater for recipients receiving embryos collected from donors treated with bovine somatotropin than from control donors (Moreira et al., 2002).

### Making a better uterus

There is considerable interest in identifying characteristics of the female that distinguish the best recipient. In women, microarray technology has been used to identify when a woman is most receptive for a transferred embryo (Gómez et al., 2015). Accurate recipient selection in cattle is a good approach when the primary goal is to ensure that a genetically valuable embryo becomes a healthy calf. However, attainment of the full benefits of the genetic and fertility-enhancing effects of ET requires that a large proportion of females receive an embryo (Kaniyamattam et al., 2018). Identifying superior recipients is not feasible in such situations. One important goal, accordingly, is to increase reproductive tract health at the herd level. Methods to achieve that goal will enhance fertility not only after ET but also for natural mating and AI.

A great deal has already been achieved in improving female fertility. Many recently developed TAI programs, which are also used for TET (Baruselli et al., 2011), not only avoid the need for detection of estrus but also enhance fertility as compared with insemination at estrus (Carvalho et al., 2018). Improvements in P/AI could reflect ovulation of a better-quality oocyte or establishment of an endocrine environment that creates a better oviductal or uterine environment for support of pregnancy. A method that improves oocyte quality or fertilization rate would not be relevant for fertility of the ET recipient. An example is the treatment of lactating cows with progesterone during the preovulatory period, which increased P/AI but not P/ET (Pereira et al., 2017). Treatments that improved endometrial function would, however, be beneficial for inseminated females and ET recipients. Hence, several hormonal treatments have been tested for effectiveness in improving recipient competence to maintain pregnancy after ET.

Progesterone is an important regulator of endometrial function during early pregnancy (Lonergan et al., 2016). Nonetheless, it is not clear whether the circulating concentration of progesterone at the time of ET is an important determinant of pregnancy success, provided the recipient has a functional CL. There was no relationship between CL size (Coleman et al., 1987; Spell et al., 2001; Pugliesi et al., 2019) or circulating progesterone concentrations (Spell et al., 2001) at the time of transfer and P/ET although blood perfusion of luteal tissue was positively related to pregnancy success (Pugliesi et al., 2019). Results have also been mixed as to whether pharmacological methods to increase progesterone in recipients can increase P/ET. Among the methods used include administration of progesterone via intravaginal devices and injection of gonadotropins or GnRH or its analogs. In some cases, positive effects on P/ET have been reported (Vasconcelos et al., 2011b; Wallace et al., 2011; Marques et al., 2012) whereas, in others, treatment was without effect (Ellington et al., 1991; Purcell et al., 2005; Niles et al., 2019; Steichen and Larson, 2019; García-Guerra et al., 2020) or had a negative effect (Monteiro et al., 2015).

Work in the 1970s and 1980s with ovariectomized ewes demonstrated that a uterine environment supportive of the development of a transferred embryo could be induced by sequential hormonal treatments to generate periods of high progesterone, high estrogen, low progesterone, and then high progesterone (Wilmut et al., 1986). Perhaps, there are opportunities to use the regulation of steroid hormones to better prepare the uterus for support of embryonic development but the existing methodologies cause changes in endocrine status of the recipient that are not precise enough to ensure an optimal reproductive tract environment in all cases.

There are reports of other hormonal treatments that improve P/ET, including injection of bovine somatotropin (Moreira et al., 2002; but see Hasler et al., 2003 for lack of an effect), feeding a supplement containing sunflower seed (Cordeiro et al., 2015), and infusion of peripheral blood mononuclear cells into the uterus at the time of transfer (Ideta et al., 2010). More work should be conducted to confirm these effects and develop other treatments that can enhance recipient receptivity.

Genetic selection has proven effective at increasing the fertility of lactating dairy cows (Cole and VanRaden, 2018). One of the components of fertility is the competence of an embryo to establish and maintain pregnancy; another is the capacity of the reproductive tract to support a developing conceptus. Continued selection for reproduction will likely result in higher P/ET as well as P/AI, even though the heritability of pregnancy success after ET is very low, being estimated at 0.03 for recipients and 0.02 for embryos (Parker Gaddis et al., 2017).

### Forging new tools for embryo production and transfer

Rienzi et al. (2011) pointed out that the devices used for embryo culture had largely been unchanged since the beginning of IVP. Since this paper was published, incubators designed specifically for embryo culture that deliver gases with low oxygen content are commercially available. Additionally, time-lapse monitoring of development is also a common feature of human-assisted reproduction laboratories (Basile et al., 2019). Otherwise, the statement made by Rienzi et al. (2011) holds largely true today for the production and transfer of embryos. Most culture systems rely on plastic Petri dishes, and the devices used to transfer embryos to recipients have remained largely unchanged in their essential features since their original development.

It does not have to be this way. Rather than simply relying on what already exists, often designed for other purposes (Petri dishes and Foley catheters), there are opportunities to devise tools that are optimized for use with embryo technologies. Some of these new tools can be simple, as for the above-mentioned example of new ET pipettes (Jaśkowski et al., 2010). Others can be much more sophisticated. Advances in computer technology, materials sciences, and tissue engineering have made it possible to build complex assemblies for the culture of cells and tissues (Alzamil et al., 2020; Morley et al., 2020; Terrell et al., 2020). Some of these systems could allow cultured gametes and embryos to experience some of the same mechanical forces, interactions with the substratum of the oviduct and endometrium, and dynamic changes in the microenvironment that occur in the reproductive tract.

**Figure 7.**
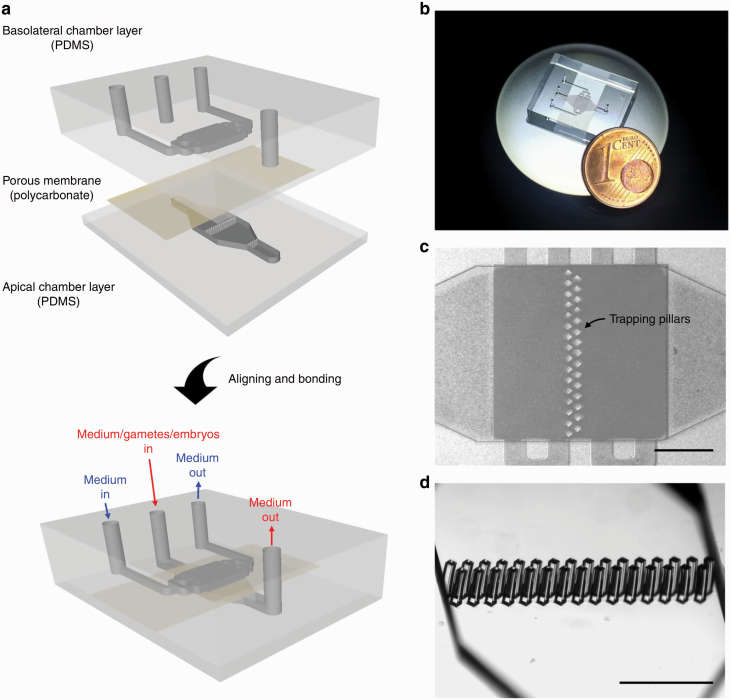
Oviduct-on-a-chip. This device was manufactured by soft lithography using two chambers constructed of polydimethylsiloxane (PDMS) and separated by a porous polycarbonate membrane (a). The basolateral chamber was designed to mimic features of the vasculature including delivery of progesterone and estradiol, while the apical chamber was designed to mimic the oviductal epithelium. Both chambers were perfused independently. Oviductal epithelial cells were grown on the apical side of the porous membrane. A photograph of the device is in (b). Movement of oocytes and embryos were arrested in the apical chamber by trapping pillars shown in (c) and (d). The figure is reproduced from Ferraz et al. (2018) and is reproduced from *Nature Communications* through a Creative Commons Attribution 4.0 International License.

One technology that has seen implementation in the field of assisted reproduction is microfluidics, for both IVP of embryos and cryopreservation (Zhao and Fu, 2017; Weng, 2019). In cattle, microfluidic devices have been developed for sorting more viable sperm (Li et al., 2016) and oocytes (Iwasaki et al., 2018). A microfluidic apparatus containing bovine oviductal epithelial cells that is illustrated in Figure 7 has been used to support fertilization and embryonic development through day 4 of development (Ferraz et al., 2018). Technical issues contributed to lower cleavage rates and rates of development than for a conventional embryo production system but DNA methylation intensity and transcript abundance in zygotes produced in the microfluidics device were more like that for embryos produced in vivo using superovulation than for embryos produced in a conventional IVP system.

### The need for markers of a fertile embryo and receptive uterus

Transfer experiments are crucial to improving the methodology for ET. Nonetheless, progress in improving P/ET will be slow unless reliable methods for estimating embryo competence for the establishment of pregnancy and female competence for supporting pregnancy not dependent upon ET can be established. This is because of the scale required to perform adequately powered experiments to evaluate whether a specific treatment can enhance pregnancy success. Consider, for example, a treatment that was hypothesized to increase P/ET from 35% to 45%. An experiment with sufficient power to detect this difference in 80% of experiments would require a sample size of 376 recipients per treatment (power calculations performed using the calculator available at https://www.stat.ubc.ca/~rollin/stats/ssize/b2.html). Few laboratories have the resources to conduct ET experiments on this scale. A search of original research papers indexed in PubMed from January 2019 through June 2020 revealed only eight papers in which experiments with more than 400 transferred embryos *total* were transferred [search terms = embryo transfer and (cow or heifer)].

#### Predicting embryo competence to establish pregnancy

The most widely used method to assess embryo quality is morphological evaluation. The International Embryo Technology Society has published standards for evaluating embryo quality with grades of 1 (excellent/good), 2 (fair), 3 (poor), and 4 (unfertilized/dead/degenerate; Stringfellow and Givens, 2010). Pregnancy rates are higher when embryos with better-quality grades are transferred for both MOET (Farin et al., 1999; Hasler, 2001; Chebel et al., 2008) and IVP (Hasler et al., 1995; Farin et al., 1999). Differences in P/ET between grades 1 and 2 embryos can be small or nonsignificant; however (Spell et al., 2001; Chebel et al., 2008) and there are likely to be many embryos whose grade does not reflect competence to establish pregnancy. In addition, there can be a great deal of variation in how technicians assign quality grades (Farin et al., 1999). Machine learning has been applied to images of bovine blastocysts to increase the accuracy of morphological assessments (Rocha et al., 2017), and this approach could prove fruitful, especially if machine learning to identify morphological features was informed by information about pregnancy outcomes after transfer.

Time-lapse monitoring of embryonic development in culture to assess the timing of specific events in development (for example, time to first cleavage, and intervals between cell divisions) has been used in human-assisted reproduction to select embryos for transfer based on morphokinetic features of individual embryos. There are several reports that pregnancy success after transfer can be increased following selection based on morphokinetics (Basile et al., 2019). In the cow too, Sugimura et al. (2017) have reported that embryos selected based on time-lapse analysis indicating short duration of first cleavage, 2 rather than more than 2 blastomeres at first cleavage, and ≥6 blastomeres at the onset of the temporary developmental arrest between the fourth (5 to 8 cells) and fifth (9 to 16 cells) cell cycle achieved a higher P/ET (16/27; 59% pregnant) than expanded blastocysts selected based on conventional morphological criteria (7/22; 32% pregnant).

One method used in human-assisted reproduction to identify embryos with high competence to establish pregnancy is to determine ploidy. Some studies report improvements in pregnancy rate by using polymerase chain reaction or next-generation sequencing to determine the number of chromosomes (Sciorio and Dattilo, 2020). One difficulty is the high incidence of mixoploidy in the preimplantation human (Popovic et al., 2020) and bovine (Viuff et al., 1999) embryo. Recently, live offspring have been produced from bovine embryos that were biopsied to determine the occurrence and paternal origin of aneuploidy using karyomapping (Turner et al., 2019).

**Figure 8.**
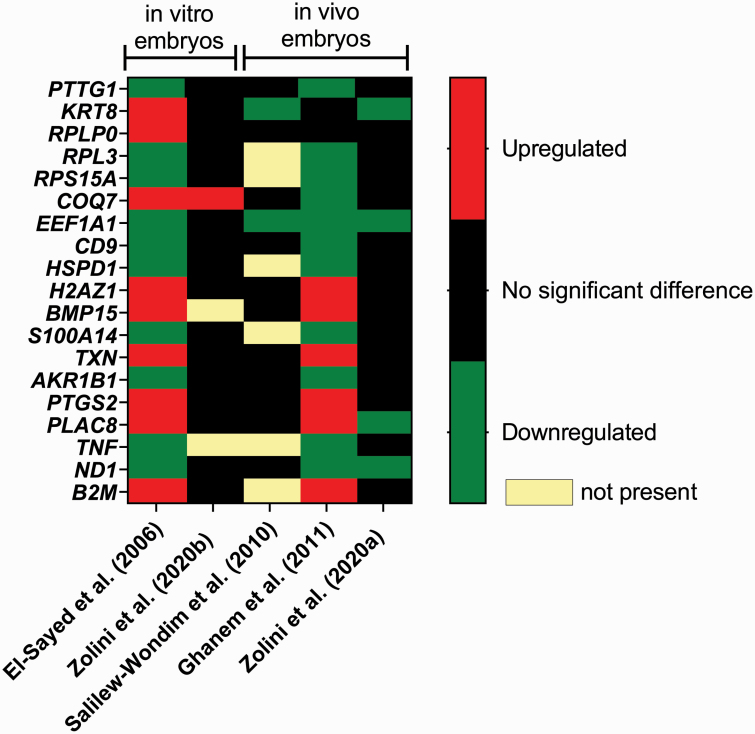
Degree of repeatability of differentially expressed genes related to embryo survival. The color code indicates whether the gene was upregulated, downregulated, or not significantly associated with either embryo survival. The figure was modified slightly from Zolini et al. (2020a) and was published with permission of *Cell and Tissue Research*.

One potential strategy for identification of embryos with competence to establish pregnancy is to establish a transcriptome signature associated with embryo survival. The approach has been to identify genes whose expression is associated with embryo survival in bisected embryos, where one demi-embryo is transferred into a recipient and the other is used for analysis of the transcriptome. This type of analysis has been performed for both MOET embryos (Salilew-Wondim et al. 2010, Ghanem et al., 2011; Zolini et al., 2020a) and IVP embryos (El-Sayed et al., 2006; Zolini et al., 2020b). In each experiment, a panel of genes was identified whose expression was associated with embryo survival after transfer. Unfortunately, there is low concordance between studies in the specific genes related to embryo survival (Figure 8). Perhaps specific characteristics of the blastocyst including breed and sex are responsible for some of the variation between experiments. Additionally, false positives are a feature of transcriptomics and their occurrence would reduce repeatability. The embryo production system is also likely to influence genes associated with embryo survival. For female Holstein embryos, many genes associated with survival of MOET embryos were related to low oxygen consumption and possible differentiation of the inner cell mass to epiblast (Zolini et al., 2020a), whereas many genes associated with survival of IVP embryos were related to cell stress and cell survival (Zolini et al., 2020b).

Two genes have been associated repeatedly with P/ET, specifically *ND1*, which was downregulated in embryos that survived after transfer, and *EEF1A1* that was downregulated in embryos that survived transfer (Figure 8). *ND1* encodes for one of the nicotinamide adenine dinucleotide dehydrogenase subunits of complex I in the electron transport chain, and *EEF1A1* encodes for the alpha subunit of the elongation factor-1 complex that participates in protein synthesis, signal transduction, organization of the cytoskeleton, and export of nuclear proteins (Sasikumar et al., 2012). Downregulation of these two genes in embryos that survived transfer is consistent with the quiet-embryo hypothesis that states that embryos are healthiest when they are not metabolically active (Leese, 2002).

#### Predicting uterine capacity for supporting embryonic development

Transcriptomics has also been used to define molecular signatures of uterine capacity for supporting embryonic development. Differences in mRNA transcript abundance have been identified at days 3, 7, 6 to 8, and 14 of the estrous cycle between heifers that subsequently became pregnant or did not after ET (Salilew-Wondim et al., 2010; Ponsuksili et al., 2012, Mazzoni et al., 2020). Differences in endometrial gene expression have also been characterized between cows that diverge in genetic estimates of fertility (Moran et al., 2017) and between fertile and infertile populations of cows identified by sequential rounds of AI (Minten et al., 2013; Killeen et al., 2014) or ET (Geary et al., 2016; Moraes et al., 2018).

Gene enrichment terms related to immune function are frequently identified when classifying differentially expressed genes in the endometrium of fertile vs. infertile females (Salilew-Wondim et al., 2010; Ponsuksili et al., 2012; Killeen et al., 2014; Moran et al., 2017; Mazzoni et al., 2020). This finding, as well as other experiments illustrating the connection between postpartum disease and infertility (Ribeiro et al., 2016) and the positive effect of intrauterine infusion of peripheral blood mononuclear cells at the time of ET (Ideta et al., 2010), points out the importance of immune function for fertility. The exact role of immune cells in the establishment and maintenance of pregnancy remains elusive but it is apparent that the specific nature of immune activation in the reproductive tract can facilitate or impede pregnancy (Hansen, 2011).

There is a compelling need to use data from these and additional new studies to identify a molecular signature of a receptive uterus. Recently, Mazzoni et al. (2020) demonstrated the utility of such an approach. Endometrial biopsies were collected from day 6 to 8 of the estrous cycle preceding a cycle in which cows received a single IVP embryo. A total of 111 genes were expressed differently between cows that became pregnant after transfer as compared with those that did not. Among the ontologies in which differentially expressed genes could be categorized were those for extracellular matrix interaction, histotroph metabolic composition, prostaglandin synthesis, transforming growth factor-β signaling, and inflammation and leukocyte activation. Subsequently, the authors used the set of differentially expressed genes and logistic regression to predict the grouping of cows from two other studies in which endometrial gene expression was determined. The accuracy of classifying endometrial samples as being from progesterone-treated or control animals (Forde et al., 2011) was 66.6%, and the accuracy of classifying animals as having high genetic potential for fertility vs. low genetic potential for fertility was 78.5% (Moran et al., 2017). These findings generate optimism that useful tools can be developed to classify cows based on the capacity to support pregnancy.

#### Can phenomics help?

The phenome is the complete phenotypic representation of a species (Mahner and Kary, 1997), and phenomics is the study of the phenome (Freimer and Sabatti, 2003). Phenomics is being emphasized as an important tool for the management and genetic improvement of crops (Mochida et al., 2020; Yang et al., 2020) and livestock (Koltes et al., 2019). As the preceding paragraphs make apparent, there is a large collection of phenotypes describing the ability of both the embryo to establish pregnancy and the female to support that pregnancy. Besides transcriptional endpoints, there are other phenotypes of a variety of organs, tissues, cells, and fluids that could be relevant to a successful pregnancy, including those derived from digital analysis of morphology and morphokinetics, physiological endpoints (e.g., behavior, color-Doppler evaluation of blood flow, and measurements of circulating hormone concentrations), and assessments of the methylome, metabolome, and lipidome. Integrating these large collections of phenotypes is likely to result in new tools for assessing embryo and maternal capacity for pregnancy and lead to improvements in both P/ET and P/AI.

## Abbreviations

AI: artificial insemination
BSA: bovine serum albumin
CL: corpus luteum
ET: embryo transfer
FSH: follicle-stimulating hormone
GnRH: gonadotropin releasing hormone
HETE: hydroxyeicosatetraenoic acid
IVP: in vitro produced (production)
MOET: multiple ovulation embryo transfer
P/AI: pregnancy per artificial insemination, expressed as a percent
P/ET: pregnancy per embryo transfer, expressed as a percent
PDMS: polydimethylsiloxane
TAI: timed artificial insemination
TET: timed embryo transfer
